# Evaluation of Surface Topography and Biomimetic Remineralization Capacity of Dendrimers in Comparison With Calcium Silicate Cement: An In Vitro Study

**DOI:** 10.7759/cureus.65812

**Published:** 2024-07-31

**Authors:** Sri Meghana Sanka, Kavitha Ramar

**Affiliations:** 1 Pediatric and Preventive Dentistry, Sri Ramaswamy Memorial (SRM) Kattankulathur Dental College and Hospital, Chennai, IND

**Keywords:** in vitro study, primary molar teeth, dentine, calcium silicate cement, dendimers

## Abstract

Introduction: Biodentine, a calcium silicate-based material, is known for its biocompatibility and ability to promote dentin regeneration. With their unique branching structure, polyamidoamine (PAMAM) dendrimers have shown promise in facilitating biomimetic remineralization processes.

Aim: This study investigates the synergistic effects of combining PAMAM with Biodentine on root dentin remineralization, aiming to develop a novel bioactive compound that offers superior protective and regenerative properties.

Methods: The following predictions were made: (1) In a cyclic artificial saliva/acid regimen, among the test groups, the combination of Biodentine and PAMAM would cause the most root dentin remineralization (2). Biodentine alone would increase Ca and P concentrations, neutralize acid, and promote root dentin remineralization (3). PAMAM, on the other hand, can remineralize the demineralized root dentin.

Results: Minimal mineral regeneration was accomplished in demineralized root dentin when treated with Biodentine or PAMAM alone. Root dentin remineralization was most pronounced when Biodentine and PAMAM were used together, and the hardness of demineralized root dentin was raised to an equivalent level to that of healthy root dentin.

Discussion: The study demonstrated the exceptional ability of PAMAM + Biodentine to promote root dentin remineralization. In an acid-challenging environment, PAMAM + Biodentine promoted full and efficient root dentin remineralization. Restorations made using innovative PAMAM + Biodentine technology show promise in remineralizing and protecting tooth structures.

## Introduction

The primary challenge faced by modern restorative dentistry is the successful remineralization of hypomineralized carious dentine so the pulp can be protected and preserved. Extrafibrillar and intrafibrillar remineralization of dentin are also possible. The residual apatite crystals in epitaxial demineralization dentin are covered by extra fibrillar remineralization [1,2]. Traditionally, pulp exposure and subsequent root canal therapy were frequent outcomes of managing deep caries. Partial caries removal techniques with a biological foundation have been promoted to prevent carious pulp exposure. The eradication of all caries, whether selectively or completely, is currently regarded as overtreatment, according to recent consensus findings [2]. Pulp vitality that results from therapeutic treatment must be preserved. Capping materials can maintain the pulp's life, functionality, and biological activity. This consistent shift is in favor of a minimally intrusive, conservative approach [3].

In the treatment approach to the carious lesion from a minimally invasive standpoint, the concept of "selective dentin removal" has superseded the traditional one of "non-selected dentin removal," which involves scraping away at tooth enamel until hard dentin is reached throughout the entire cavity. In the second, the amount of soft dentin that is removed depends on the cavity's depth; what remains is firm, leathery dentin, which can be treated appropriately to promote remineralization [4].

Histologically, dentine that is leathery and hard is the same as partly mineralized dentin, sometimes known as "affected dentin" [5]. The collagen fibers remain intact with their characteristic bands, and the hydroxyapatite crystals are shorter [6]. When teeth remineralize, they recover some of the calcified material that was lost during the demineralization process. Traditional methods involve adding varying amounts of fluoride to solutions that already contain calcium and phosphate ions. Crystals of hydroxyapatite form in dentin that have been partially demineralized, causing this to happen [7].

The bioactive substance known as Biodentine is based on pure silica cement and is primarily made of tricalcium silicate, calcium carbonate, and zirconium dioxide [8]. Calcium chloride, which is present in the liquid, takes approximately 12 minutes to harden [5]. Active Bio-Silicate Technology eliminates heavy metals such as calcium sulfate and aluminate and then releases calcium ions with a good pH for remineralization [6]. The calcium silica concentration in Biodentine, which has a small particle size and compressive strength comparable to dentin, can induce a non-classical remineralization process. Dentin and the Biodentine layer combine to generate a micro-mechanical tag that is rich in minerals. Additionally, Biodentine has been demonstrated to promote the secretion of TGF-1 by pulp cells, which can stimulate angiogenesis, cell differentiation, and mineralization processes [7].

Polyamidoamine (PAMAM) dendrimer was an additional material of preference for remineralizing tooth lesions [8,9]. To stimulate tooth remineralization in dental structures, various types of dendrimers were utilized as nucleation templates [9]. PAMAM is a superior nucleation template and has a quick absorption rate for Ca and P ions, which leads to remineralization [8,10]. Our objective in this study was to compare the efficacy of Biodentine, PAMAM, and a combination of the two in promoting dentin remineralization in the roots.

## Materials and methods

Type of study

This controlled laboratory study aimed to simulate carious lesions in primary teeth and evaluate the remineralization potential of different materials and their combinations. The experimental setup enabled precise quantification and comparison of the effects of Biodentine and PAMAM. Ethical approval was obtained from the institutional committee (IEC-8295).

Procedural methodology

PAMAM and Biodentine Synthesis

PAMAM and Biodentine were procured commercially. Septodont Biodentine (Saint-Maur-des-Fossés, France) was used in a powder-to-liquid ratio of 1:1, and PAMAM generation 0 (PAMAM G_0_) (Dendritech, Midland, Michigan) was used.

Preparation of Root Dentin Specimens

**Figure 1:**
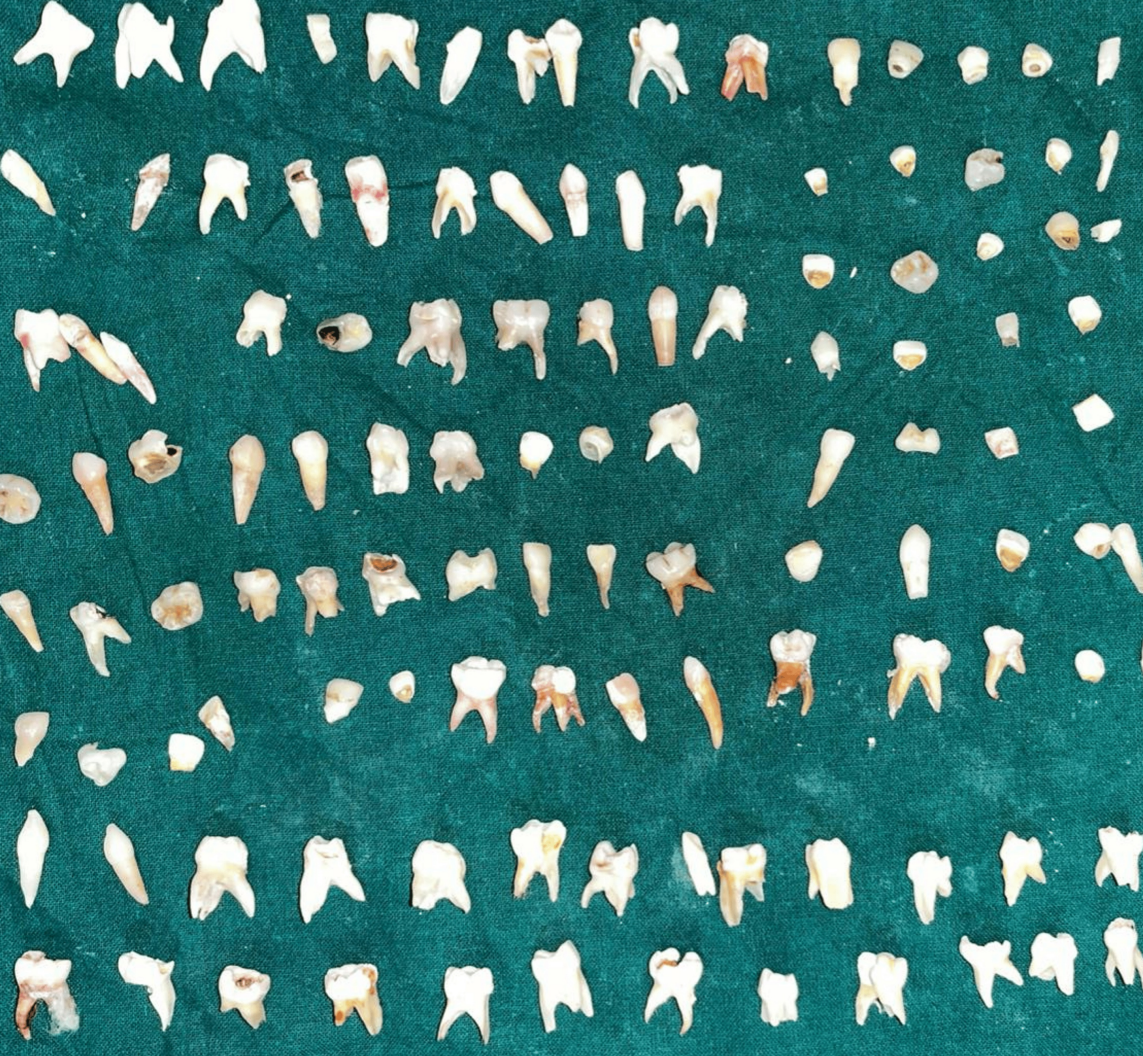
A total of 120 primary teeth were collected and sectioned into caries-free root dentine slices.

**Figure 2:**
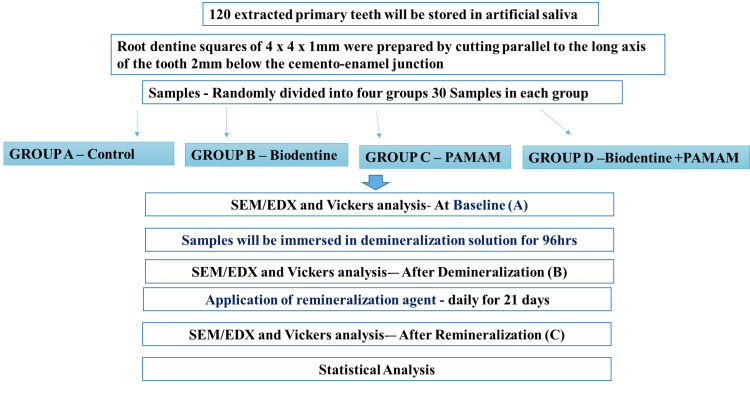
Flow chart representing the methodology of the study. Control group: absence of a remineralization agent (samples are stored in artificial saliva) SEM-EDX: scanning electron microscopy (SEM) with energy-dispersive X-ray analysis (EDX); PAMAM: polyamidoamine

The dental school clinics were scoured for primary teeth. The next step was to disinfect and store the teeth in distilled water and at room temperature (Figure 1). Using a diamond blade, 4 × 4 × 1 mm root dentin squares were created by cutting in a straight line parallel to the tooth's long axis, 2 mm below the cementoenamel junction. These dentine squares were devoid of caries. Following an earlier investigation, the root dentin samples were preserved in a demineralization solution for 24 hours (Figure 2) [11].

Remineralization of Root Dentin in an Acidic Setting

Dentin samples from demineralized teeth were split into four equal groups: Group One/Control group ensures accuracy; we immersed each root dentin specimen in distilled water and subsequently allowed it to air dry [12]. Group Two: Biodentine: three Biodentine bars of 2 × 2 × 12 mm were placed in contact with each demineralized root dentin specimen [13]. Group Three: PAMAM: to make sure the PAMAM macromolecules were immobilized on the root dentin, 100 µL of the solution was applied to each demineralized root dentin specimen. After one hour, the specimen was washed with water to remove any loose PAMAM [13]. Group Four: PAMAM and Biodentine: after 100 µL of PAMAM solution was applied to each demineralized root dentin specimen, three 2 × 2 × 12 mm Biodentine bars were positioned on top of the dentin specimen.

There were 30 samples tested for each group. The artificial saliva solution was made by mixing 1.5% CaCl_2_, 0.9% KH_2_PO_4_, 130 mmol/L KCl, 1 mmol/L NaN_3_, and 20 mmol/L 4-(2-hydroxyethyl)-1-piperazineethanesulfonic acid in distilled water. The pH was then adjusted to 7.0 using potassium hydroxide (1 mmol/L). One option for remineralization is to use artificial saliva, which mimics human saliva and delivers ions of calcium and phosphorus. The specimens in the four groups were submerged in 1 mL of new artificial saliva, which was changed after 24 hours. This regime was followed for 21 days.

Scanning Electron Microscopy (SEM) Analysis

**Figure 3:**
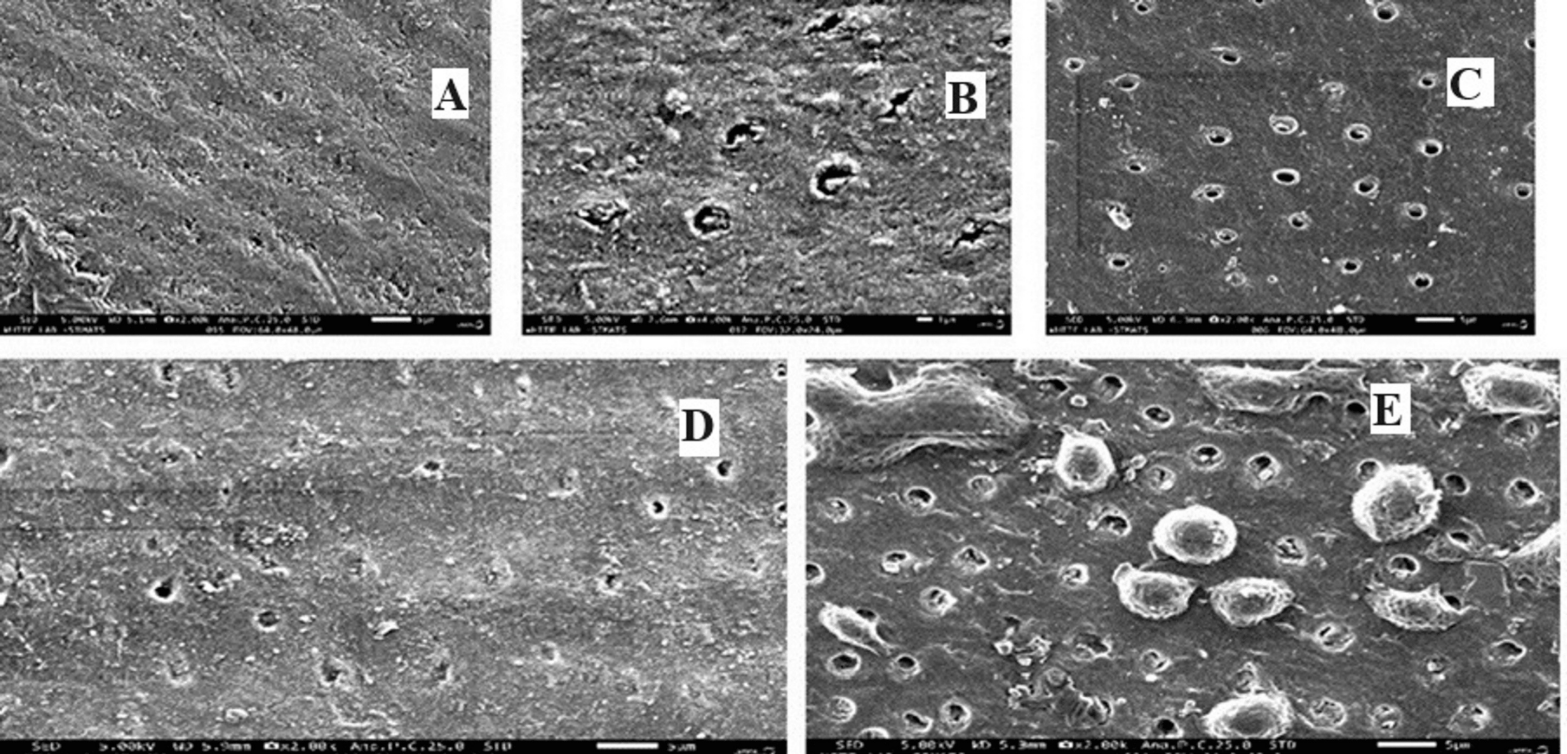
Root dentin and representative SEM pictures of (A) sound tooth, (B) demineralized tooth, (C) Biodentine group, (D) PAMAM group, and (E) PAMAM + Biodentine group. There were collagen fibrils that were found exposed in (B). The root dentin surface was seen to have regenerated minerals precipitate (C-E). In (D, E), the dentin was more heavily coated with remineralized mineral crystals, and minerals partially obstructed the dentinal tubules (C) SEM: scanning electron microscope; PAMAM: polyamidoamine

The demineralized root dentin surface and dentinal tubules were examined for mineral regeneration after 21 days by removing all root dentin squares from the solutions [14]. After that, SEM was used to evaluate the root dentin samples that had been sputter-coated with gold (JEOL 5300, Peabody, Massachusetts) (Figure 3).

Ca and P Ion Concentration Measurement

**Figure 4:**
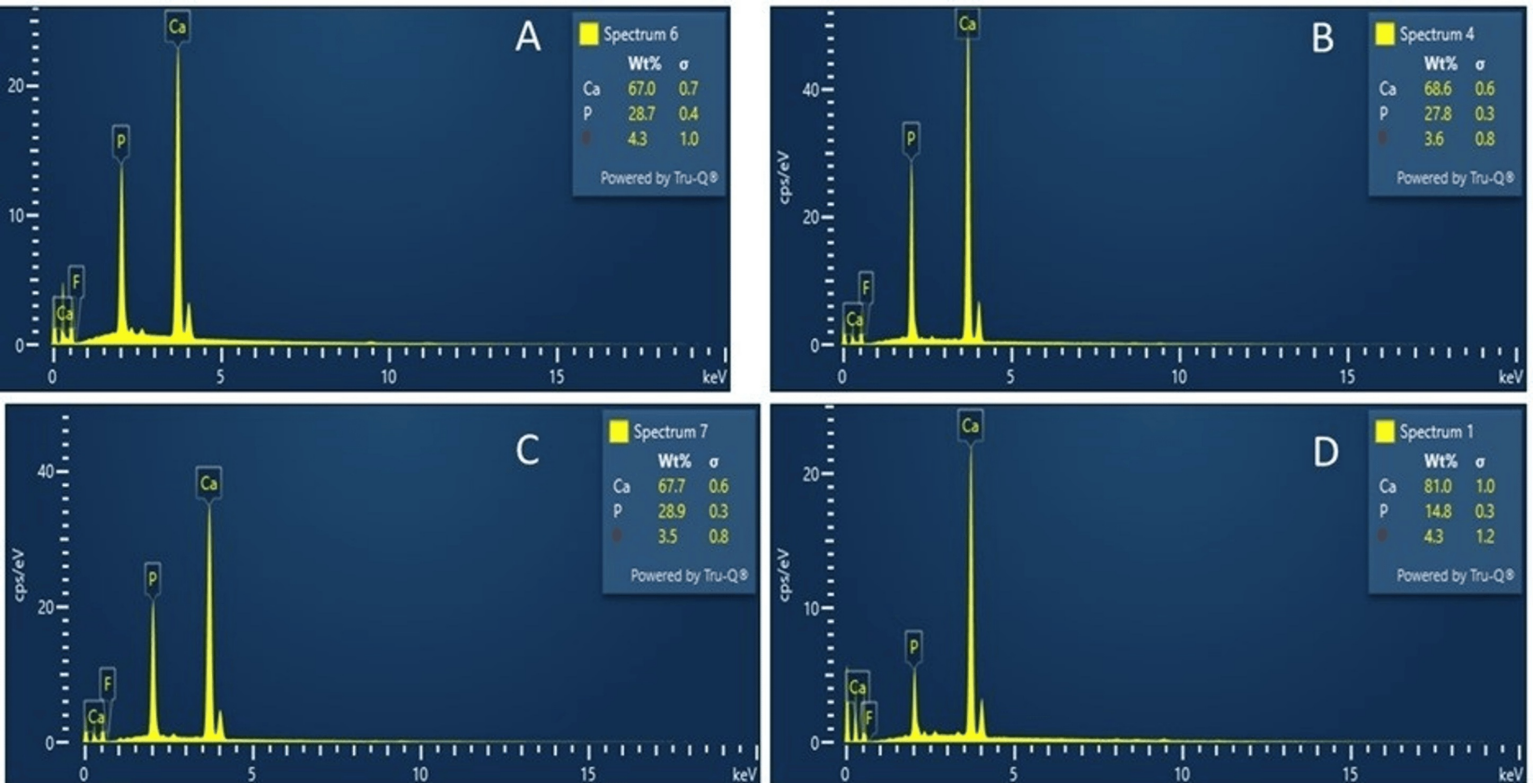
The concentrations of Ca and P ions in the (A) control group, (B) Biodentine group, (C) PAMAM group, and (D) PAMAM + Biodentine group. The Biodentine group and the PAMAM + Biodentine group had considerably increased Ca and P concentrations in artificial saliva compared to the PAMAM and control groups PAMAM: polyamidoamine

Ca and P ion concentrations were measured at the end of 21 days and before placing the samples in remineralization solution (after demineralization) and compared with sound root dentin. The collected solution was analyzed for ion concentrations via a spectrophotometric method using known standards and calibration curves, following previous studies (Figure 4) [14].

Dentin Hardness Measurement

**Figure 5:**
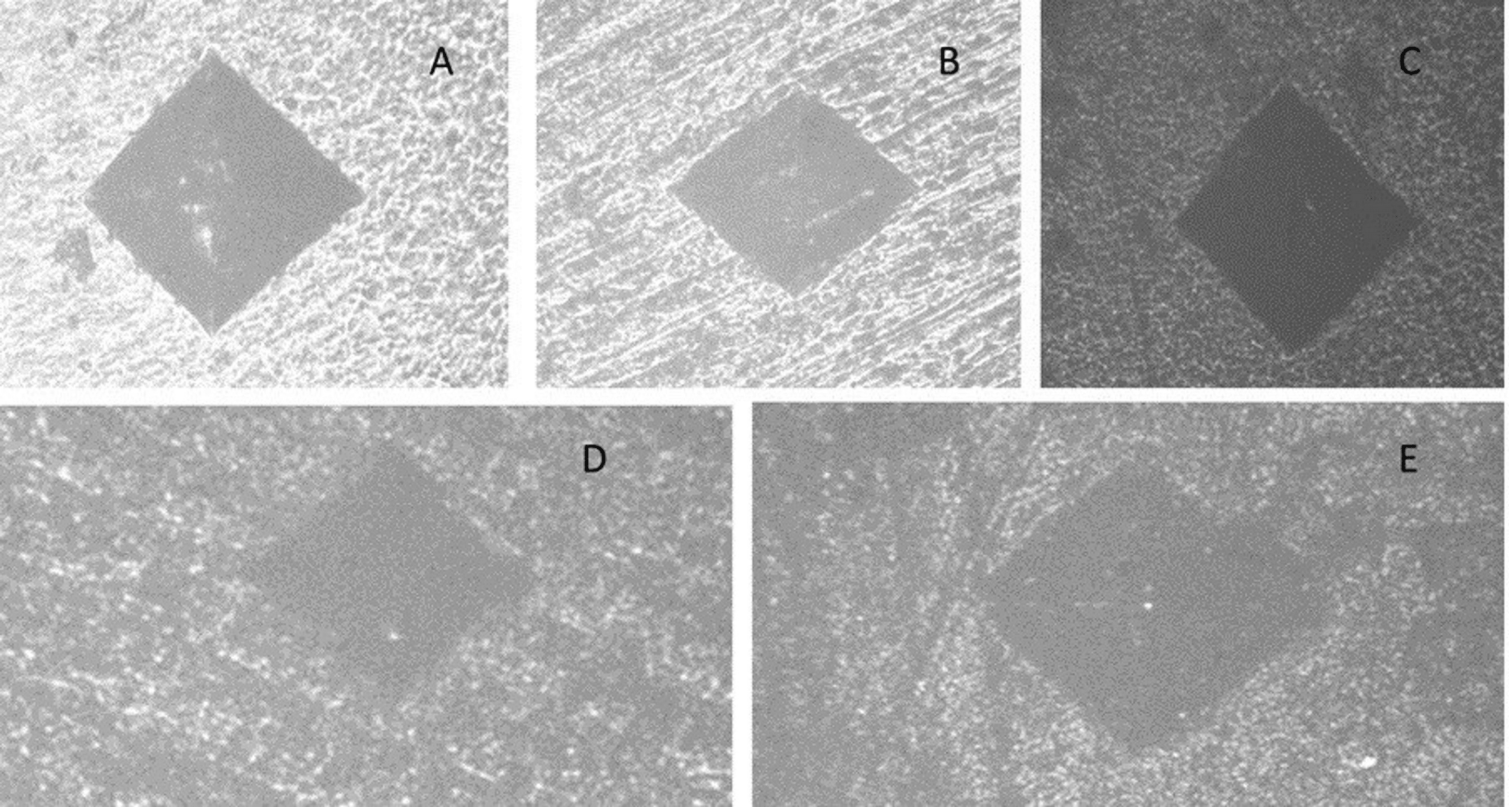
The dentin hardness of the four groups was evaluated: (A) sound tooth, (B) control group, (C) Biodentine group, (D) PAMAM group, and (E) PAMAM+ Biodentine group PAMAM: polyamidoamine

The number of minerals in the dentin structure was found to be proportional to dentin hardness; thus, surface hardness was typically used to evaluate the level of remineralization [15]. At the end of the 21st day, all four groups had their dentin hardness evaluated. The Vickers diamond indenter was employed with a Tukon 2100B hardness tester, with a load of 20 g and a dwell period of 10 seconds [16]. For each group, 10 dentin specimens were tested after three indentations were formed in the dentin. Additionally, dentin hardness values were measured before and after acid-etching, as well as after the 21-day regime, to serve as comparison controls (Figure 5).

Statistical analysis

IBM SPSS Statistics for Windows, Version 24 (Released 2016; IBM Corp., Armonk, New York) was utilized to conduct a statistical analysis of the acquired values. The data were analyzed using the Turkey Multiple Comparison Test. At the p<0.05 threshold, the significance was demonstrated.

## Results

Table 1 provides a detailed statistical summary of Vickers hardness test results across six different groups: sound, demineralized, control, Biodentine, PAMAM, and PAMAM + Biodentine. Each group contains 10 values. The minimum hardness values observed are 62.9 for the sound group, 26 for demineralized, 23.2 for control, 40 for Biodentine, 40.3 for PAMAM, and 43.8 for combination. The maximum hardness values recorded are 65 for the sound group, 30 for demineralized, 29.5 for control, 43.8 for Biodentine, 45 for PAMAM, and 48.6 for combination. The ranges, which are the differences between the maximum and minimum values, are 2.1, 4, 6.3, 3.8, 4.7, and 4.8 for each group, respectively.

**Table 1:** A comprehensive overview of the descriptive statistics for all the groups. PAMAM: polyamidoamine

Parameters	Sound	Demineralized	Control	Biodentine	PAMAM	Combination
Number of values	10	10	10	10	10	10
Minimum	62.9	26	23.2	40	40.3	43.8
Maximum	65	30	29.5	43.8	45	48.6
Range	2.1	4	6.3	3.8	4.7	4.8
Mean	63.99	27.89	26.8	41.72	43.11	45.73
Std. deviation	0.7109	1.345	2.207	1.195	1.513	1.43
Std. error of the mean	0.2248	0.4254	0.6979	0.3779	0.4785	0.4522

The mean hardness values, which represent the average hardness for each group, are 63.99 for the sound group, 27.89 for demineralized, 26.8 for control, 41.72 for Biodentine, 43.11 for PAMAM, and 45.73 for combination. The standard deviations, indicating the amount of variation or dispersion from the mean, are 0.7109, 1.345, 2.207, 1.195, 1.513, and 1.43, respectively. The standard errors of the mean, which measure the accuracy with which the sample mean represents the population mean, are 0.2248 for the sound group, 0.4254 for demineralized, 0.6979 for control, 0.3779 for Biodentine, 0.4785 for PAMAM, and 0.4522 for combination. These statistics provide a comprehensive overview of the hardness properties of each group, highlighting differences and variations among them.

The results of Dunn's multiple comparisons test (Tables 2, 3) reveal significant differences in Vickers hardness values among the various material groups compared to the control group. When comparing the control group to the sound group, there is a significant mean rank difference of -46.35, with an adjusted p-value of less than 0.0001, indicating a highly significant result (****). Similarly, the comparison between the control and combination groups also shows a highly significant mean rank difference of -35.45, with an adjusted p-value of less than 0.0001 (****). The control versus PAMAM comparison reveals a significant mean rank difference of -24.9, with an adjusted p-value of 0.0071 (**), indicating statistical significance. However, the comparisons between the control group and the demineralized and Biodentine groups do not show significant differences. The mean rank difference for control versus demineralized is -2.7, with an adjusted p-value greater than 0.9999 (not significant). For control versus Biodentine, the mean rank difference is -18.7, with an adjusted p-value of 0.0832 (not significant). These results highlight that while some material groups, specifically the sound, PAMAM, and combination, show significant differences in hardness compared to the control, other groups, such as the demineralized and Biodentine groups, do not exhibit such differences.

**Table 2:** The results of the mean rank difference for Dunn's multiple comparisons test. PAMAM: polyamido amine; n1: sample size in Group 1; n2: sample size in Group 2

Test Details	Mean Rank 1	Mean Rank 2	Mean Rank Difference	n1	n2
Control vs. sound	9.15	55.5	-46.35	10	10
Control vs. demineralized	9.15	11.85	-2.7	10	10
Control vs. Biodentine	9.15	27.85	-18.7	10	10
Control vs. PAMAM	9.15	34.05	-24.9	10	10
Control vs. combination	9.15	44.6	-35.45	10	10

**Table 3:** The results of Dunn's multiple comparisons test provide a detailed comparison of the control group with each of the other groups (sound, demineralized, Biodentine, PAMAM, and combination). *denotes a significant difference A: sound; B: demineralized; C: control; D: Biodentine; E: PAMAM; and F: PAMAM and Biodentine (combination group) PAMAM: polyamidoamine; ns: not significant

Dunn's Multiple Comparisons Test	Mean Rank Difference	Significant	Summary	Adjusted P-value	Combination Group
Control vs. sound	-46.35	Yes	****	<0.0001	A
Control vs. demineralized	-2.7	No	ns	>0.9999	B
Control vs. Biodentine	-18.7	No	ns	0.0832	D
Control vs. PAMAM	-24.9	Yes	**	0.0071	E
Control vs. combination	-35.45	Yes	****	<0.0001	F

## Discussion

The present study marks the first instance of combining PAMAM with Biodentine to address tooth caries. This innovative approach resulted in significant root dentin remineralization. This new method successfully remineralized demineralized root dentin in a cyclic artificial saliva environment. The hypotheses were confirmed, demonstrating that adding PAMAM did not compromise mechanical properties and that the PAMAM-Biodentine combination was the most effective for inhibiting demineralization and promoting remineralization of tooth root dentin.

The development of minimally invasive, biologically based therapies aimed at preserving pulp vitality remains a key focus within modern clinical endodontics [15]. Previous research indicates that both MTA and Biodentine are reliable materials for inducing dentin bridge formation while maintaining pulp vitality in both direct and indirect pulp-capping procedures. Biodentine, compared to MTA, offers easier handling, lower cost, and faster setting times, with comparable or superior clinical outcomes [16]. It also boasts high biocompatibility and excellent bioactivity, although more long-term clinical studies are needed for a definitive evaluation of Biodentine as a pulp-capping agent [17]. Recent studies have also shown Biodentine's excellent antimicrobial activity upon initial contact with bacteria [18].

During remineralization, PAMAM attracts calcium ions through its numerous amine and amide groups, forming complexes that facilitate mineral deposition [19]. Previous studies have highlighted PAMAM's excellent antibacterial properties. This study focused on developing new restorative material and evaluating the remineralization ability of tooth root dentin [20]. Most previous remineralization studies were conducted in neutral pH solutions. However, acids produced by oral biofilms or acidic foods can lower the local pH significantly. Artificial saliva is commonly used for remineralization studies, with previous research employing a demineralization solution at pH 4 [21].

Dentin hardness depends on the calcified matrix amount and tubular density. It serves as an indirect measure of mineral loss or gain in dental hard tissues. Surface hardness recovery indicates successful remineralization. However, the hydration and dehydration of tissues can affect dentin's mechanical properties, necessitating evaluations of fully hydrated tissues. Previous studies by Grech et al. demonstrated that Biodentine exhibited superior microhardness compared to other materials [22]. Camilleri et al.'s research also showed that Biodentine had a higher surface microhardness than conventional and resin-modified glass ionomers [23].

The pH and calcium and phosphate ion release results were consistent with previous findings, showing that Biodentine rapidly increased the release of these ions, aiding in acid neutralization and mineral deposition. In the control group, demineralization continued, and root dentin hardness decreased [24]. The demineralized root dentin alone could not induce effective remineralization due to its weak nucleation ability and lack of acid-neutralization capabilities. Further demineralization and decreased dentin hardness were also partly due to the damage of the remaining collagen matrix by activated matrix metalloproteinases and cysteine cathepsins [25].

The Biodentine group exhibited moderate remineralization, with mineral precipitation in root dentin and greater hardness than the control group. Biodentine's release of calcium and phosphate ions benefited remineralization. The PAMAM group showed similar remineralization effects through a different mechanism, attracting calcium and phosphate ions to facilitate rapid mineral regeneration [26]. In contrast, the novel PAMAM + Biodentine combination showed the most significant mineral regeneration and root dentin hardness, reaching the level of healthy dentin. This combination provided triple benefits: excellent nucleation templates, high ion concentrations, and acid-neutralization [27]. The results demonstrate that the PAMAM + Biodentine method effectively inhibited demineralization and promoted remineralization of root dentin.

The PAMAM + Biodentine strategy could be applied to various dental procedures, such as filling the base of cavities with Biodentine and coating with PAMAM to protect surrounding root dentin. Biodentine is already a promising material for restorations and pulp-capping [28]. The combined use of Biodentine and PAMAM offers benefits for tooth caries protection and is potentially applicable to various types of cavity restorations.

However, there are limitations to the study. Although In vitro studies provide valuable initial insights, they do not fully replicate the complex biological environment of the human oral cavity. Future studies should include in vivo experiments to confirm the efficacy and safety of the PAMAM and Biodentine combination under clinical conditions. The study's timeframe for evaluating remineralization may not be sufficient to understand the long-term effectiveness and durability of the PAMAM and Biodentine combination. Future research should focus on longer-term studies to evaluate the persistence of the remineralization effects and potential degradation of the materials.

## Conclusions

This study developed a novel bioactive material combining remineralization, protein-repellent, and antibacterial capabilities for restorative purposes. The effects of PAMAM and Biodentine on the remineralization of demineralized root dentin in a cyclic artificial saliva environment were investigated for the first time. The PAMAM + Biodentine combination was identified as the most effective method for inhibiting demineralization and promoting remineralization of root dentin, restoring the hardness to the level of healthy dentin. This method promises a wide range of dental applications to combat caries and protect tooth structures.
